# Biological and mechanical performance of calcium phosphate cements modified with phytic acid

**DOI:** 10.1007/s10856-024-06805-y

**Published:** 2024-06-20

**Authors:** Valentin C. Steinacker, Jan Weichhold, Tobias Renner, Sebastian Gubik, Andreas Vollmer, Niko Breitenbücher, Andreas Fuchs, Anton Straub, Stefan Hartmann, Alexander C. Kübler, Uwe Gbureck

**Affiliations:** 1 Department of Oral & Maxillofacial Plastic Surgery, University Hospital Würzburg, Pleicherwall 2, 97070 Würzburg, Germany; 2 Department for Functional Materials in Medicine and Dentistry, University Hospital Würzburg, Pleicherwall 2, 97070 Würzburg, Germany

## Abstract

Calcium phosphate cements, primarily brushite cements, require the addition of setting retarders to ensure adequate processing time and processability. So far, citric acid has been the primary setting retarder used in this context. Due to the poor biocompatibility, it is crucial to explore alternative options for better processing. In recent years, the setting retarder phytic acid (IP6) has been increasingly investigated. This study investigates the biological behaviour of calcium phosphate cements with varying concentrations of IP6, in addition to their physical properties. Therefore cytocompatibility in vitro testing was performed using osteoblastic (MG-63) and osteoclastic (RAW 264.7 differentiated with RANKL) cells. We could demonstrate that the physical properties like the compressive strength of specimens formed with IP6 (brushite_IP6_5 = 11.2 MPa) were improved compared to the reference (brushite = 9.8 MPa). In osteoblast and osteoclast assays, IP6 exhibited significantly better cytocompatibility in terms of cell activity and cell number for brushite cements up to 11 times compared to the brushite reference. In contrast, the calcium-deficient hydroxyapatite (CDHA) cements produced similar results for IP6 (CDHA_IP6_0.25 = 27.0 MPa) when compared to their reference (CDHA = 21.2 MPa). Interestingly, lower doses of IP6 were found to be more effective than higher doses with up to 3 times higher. Additionally, IP6 significantly increased degradation in both passive and active resorption. For these reasons, IP6 is emerging as a strong new competitor to established setting retarders such as citric acid. These cements have potential applications in bone augmentation, the stabilisation of non-load bearing fractures (craniofacial), or the cementation of metal implants.

**Graphical Abstract:**

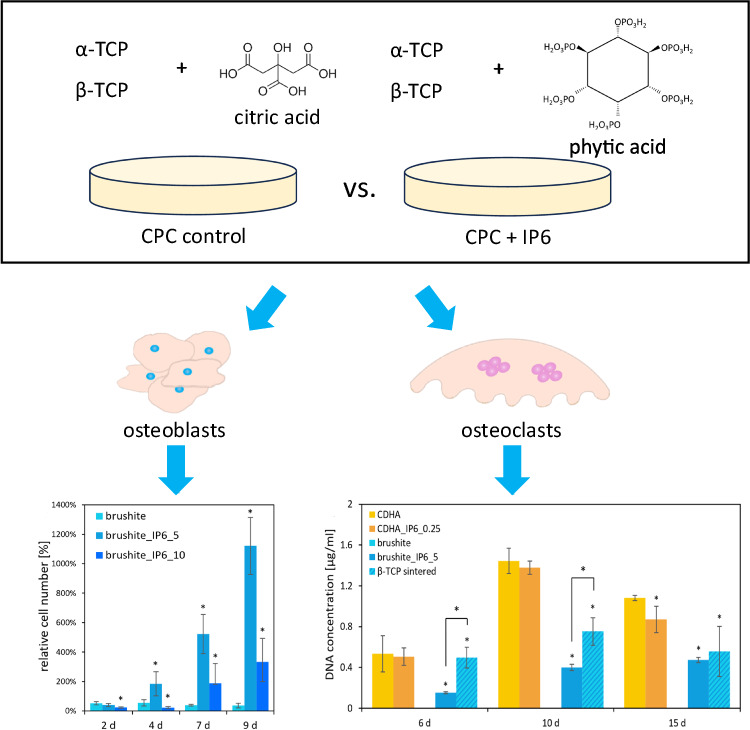

## Introduction

A major obstacle in clinical traumatology, tumour surgery and surgery of bone malformation is the critical size of the bone defect at which implantation of a bone graft becomes necessary. These grafts need to meet standards like injectability, high compressive strength, and easy processing. When cements are applied in load-bearing areas, such as in the fixation of an endoprosthesis, the mechanical requirement for compressive strength is 70 MPa [1]. Additionally, they must meet biological requirements, such as good cytocompatibility, osteoinduction, or inhibition of fibrous tissue ingrowth [2, 3]. Besides autograft and allograft, the use of synthetic bone graft provides a near endless supply of substitute material. Unlike allografts, synthetic grafts are easier to maintain consistent quality and there is no risk of transmission of infectious or malignant properties of a donor. However, infections of any graft, for example due to mishandling or bacteraemia, are a real problem [4].

Commonly used synergetic materials are calcium phosphate cements such as apatite and brushite, which can be used as sintered material [5], as granulate [6] for bone augmentation and preformed moulds or as self-setting cement systems, that can be injected directly into bone defects [7, 8].

Due to their varying characteristics, apatite-based cements are more commonly used for their mechanical strength, whereas brushite based cements present less mechanical strength but show a much higher resorbability [9]. For the use of these cements in clinical routine processability and injectability are important factors. On the one hand, the material has to allow sufficient time for application and moulding, but on the other hand, there must be no delay in the operation. This can be accomplished by varying the liquid powder ratio or using cement powders of smaller particle size [10]. However, these alterations of the educts can lead to a decrease of the compressive strength of the cements [11].

Another possibility to enhance the injectability and processability is to prolong the setting reaction by adding setting retarders. For clinical use of brushite-based cements, the addition of setting retarders is necessary due to the very fast setting reaction [12]. Citric acid, commonly used as a setting retarder, not only delays the setting reaction but also improves the physical properties of the brushite such as compressive strength and injectability [13, 14]. The drawback of citric acid is the decrease in cytocompatibility, like a decrease in osteoblastic and osteoclastic cell-numbers and cell activity [15]. This could be partly linked to a loss of cell attachment in 3T3 fibroblast cell line due to the formation of a dicalcium phosphate citrate complex [16]. A further point is the cement degradation in vivo [17]. Those additives act as chelating agents by binding calcium ions such as in α-tricalcium phosphate or β-tricalcium phosphate, which alters the growth of the crystal structure of brushite [18] and apatite [19–21]. These initial setting times vary around 50 s for brushite [15, 22] and 5 min for CDHA [23] cements without setting retarder.

For brushite cements the use of phytic acid (IP6) is described as an alternative setting retarder. Meininger et al., as part of our research group, were already able to demonstrate a comparable retardation in the setting reaction of brushite with 0.5 M citric acid and 0.1 M IP6 of >5 min and 4 min at a powder to liquid ratio of 3 g/ml [15].

Even if no setting retarders are required for an average processing time for CDHA cements, our research group was able to demonstrate significantly improved injectability of these cements in a previous study. This improved to 80% for CDHA with 0.25 wt% and 0.5 wt% IP6 up to 10 min after mixing compared to the reference without IP6 with an injectability of 20–30% [24]. Again, our research group was able to demonstrate increased injectability of over 85% for brushite cements with IP6 from 5 wt% [25]. In addition to the improvement in the physical properties of cements through IP6, initial improvements in cytocompatibility were also evident [15]. The use of IP6 in apatite cements is a relatively recent development.

In this study we wanted to investigate the mechanical strength of apatite and brushite cements produced with different amounts of IP6 after 7 d of storage. Since the cytocompatibility of CDHA cements with IP6 as setting retarder has not yet been investigated, the focus of this study is on the investigation with the MG-63 and RAW 264.7 cell lines compared with the cytocompatibility of brushite cements with IP6 as setting retarder. We also tested the cements for degeneration through osteoclast activity, which leaves room for new bone formation when used as bone graft. A comprehensive understanding of cytocompatibility is essential for the targeted development of bone cements intended for routine clinical use.

## Materials and methods

### Powder fabrication

For the synthesis of α-tricalcium phosphate (α-TCP), a mixture comprising monetite (CaHPO_4_, Honeywell, USA) and calcite (CaCO_3_, Merck, Germany) in a molar ratio of 2:1 underwent mechanical milling using a planetary ball mill (PM 400, Retsch, Germany) for 1 h. Subsequently, the resulting powder blend was subjected to sintering at 1400 °C for 5 h. Following sintering, the powder underwent further refinement through a 1 h milling process in the planetary ball mill.

Apatite was synthesised through the stirring of α-TCP suspended in 1 l of deionized water, supplemented with 30 ml of a 2.5% Na_2_HPO_4_ solution. After a period of 7 d, the suspension underwent filtration, and the obtained powder was dried at 60 °C in an oven.

The fabrication of β-tricalcium phosphate (β-TCP) involved the utilisation of monetite and calcite at a molar ratio of 2.15:1. Sintering was conducted at 1050 °C for 5 h in a specialized sintering furnace. The resultant β-TCP sintering cake was further processed by crushing with a pestle and mortar, followed by a 1-h dry milling procedure using a planetary ball mill.

### Sample fabrication

The brushite paste compositions consisted of three solutions of phosphoric acid 85% H_3_PO_4_ (Merck, Darmstadt, Germany) with either no IP6 or 5 wt% or 10 wt% of IP6 (C_6_H_18_O_24_P_6_, Sigma Aldrich, Steinheim, Germany) referred to the amount of β-TCP. The IP6-free cement instead contained 0.5 M citric acid (C_6_H_8_O_7_, Sigma Aldrich, Steinheim, Germany), as processing without setting retarders was not possible. The cement specimens were formed with a liquid powder ratio of 0.5 ml/g through homogeneous mixing on a glass slab and labelled as brushite, brushite_IP6_5 and brushite_IP6_10.

For the CDHA formulation, the powder was composed of 90 wt% α-TCP and 10 wt% CDHA, with either no IP6 or 0.25 wt% or 0.5 wt% IP6, which was added related to the mass of the cement powder. The CDHA specimens obtained a liquid to powder ratio of 0.3 ml/g of a 0.2 M disodium phosphate solution to the milled powder and were prepared in the same way as the brushite cements and then labelled CDHA, CDHA_IP6_0.25 and CDHA_IP6_0.5.

The cement pastes were moulded in silicon forms and hardened for 7 d at 37 °C and 100% humidity.

### Characterisation

The specimens for the compressive strength consisted of cuboids measuring 6 × 6 × 12 mm³. These were tested using the universal testing machine Z010 (Zwick, Germany) with a crosshead speed of 1 mm/min. For each formulation, testing was performed with 8 samples.

The phase compositions of the synthesised powders and the brushite and CDHA specimens were verified using X-ray diffractometry (XRD), which was performed using a D8 Advance with a DaVinci design diffractometer (Bruker AXS, Karlsruhe, Germany). Therefore, an angle range of 7° to 70° (2θ) was measured with a step size of 0.0112° and an integration time of 0.2 s using copper K_α_ radiation.

### Biological testing

The specimens for biological testing were formed as discs with a diameter of 5 mm and a height of 2 mm for incubation with RAW 264.7 and discs with a diameter of 15 mm and a height of 2 mm for incubation with MG63. To investigate the pH development of the hardened specimen, the pH value of the PBS washing solution was checked every hour with a InoLab Level 1 pH-meter (Xylem Analytics Germany Sales GmbH & Co. KG, WTW, Weilheim, Germany) until a physiological pH value was achieved.

For biological testing, all scaffolds were γ-sterilised by BBF Sterilisationsservice GmbH (Kernen, Germany) with a dose of 32.6 kGy.

The osteoblast-like MG-63 cell line (ATCC no. CRL-1427, Rockville, MD, USA) was cultured in Dulbecco’s Modified Eagle’s Medium (DMEM, Gibco, Cat. No.: 31966-021) supplemented with 10% foetal calf serum (FCS, Gibco, Cat. No.: 10270-106) and 1% Penicillin-Streptomycin (Gibco, Cat. No.: 15140-122). The specimens (*n* = 4) were placed in 24-well plates with sterile forceps, with the polystyrene surface as the control. To set the specimens, they were incubated for 24 h with DMEM and subsequently seeded with 5 × 10^4^ cells/well. A CASY 1 cell analyser (Schärfe System, Reutlingen, Germany) was used to perform cell counting. The plates were then incubated at 37 °C and 5% CO_2_. Cell activity and cell number were then analysed on days 2, 4, 7 and 9. For this, samples were incubated for 30 min with WST-1 reagent at a 1:10 dilution. The activity was then measured in duplicates using a microplate reader (Tecan Spark® 20 M, Tecan, Maennedorf, Switzerland). The cell number was determined again using the Casy cell counter, after the cells were detached from the surface of the samples by incubating them for 12 min with Accutase (Sigma Aldrich, A6964).

In addition, to study the effect of setting retarders on osteoclastic cells, the murine cells of Raw 264.7 (ATCC no. TIB-71, Rockville, MD, USA) were treated with Dulbecco’s Modified Eagle’s Medium (DMEM, Gibco, Cat. No.: 31966-021) which was supplemented with 10% foetal calf serum (FCS, Gibco, Cat. No.: 10270-106) and 1% Penicillin-Streptomycin (Gibco, Cat. No.: 15140-122). The cells were used up to passage 12. The specimens (*n* = 4 for quantitative TRAP and DNA testing; *n* = 2 for TRAP staining and SEM) were placed in 96-well plates and seeded with 2 × 10^4^ cells/well. The cell count was also determined with the CASY cell counter. Differentiation was achieved by adding of 50 ng/ml RANKL (R&D systems, Cat. No.: 462-TEC). The change of the medium with 50 ng/ml RANKL was performed every 48–72 h. Each sample was measured twice and the quantifiable data was determined as the mean value with its standard deviation. Moreover, the statistical analysis was performed as *t*-test.

Tartrate-resistant acid phosphatase (TRAP) is a specific marker for osteoclastic differentiation. The intracellular TRAP activity was established on lysed RAW 264.7 cells. Therefore, the surfaces were repeatedly rinsed with PBS, the specimens (*n* = 4) were transferred to a new plate and then incubated in 500 µl 1% Triton X-100 (Sigma-Aldrich, Steinheim, Germany) on ice for 60 min. For preservation, the lysates were then frozen at −80 °C. TRAP was quantified by determining the conversion of p-nitrophenyl phosphate (pNPP) to p-nitrophenol (pNP). To 50 µl lysate, 150 µl of substrate solution (100 mM sodium acetate, 50 mM disodium tartrate dehydrate and 7.6 mM p-nitrophenyl phosphate disodium hexahydrate (pNPP)) were added and incubated for 60 min at 37 °C and 5% CO_2_. 50 µl of 3 M NaOH was added to stop the enzyme reaction and the absorbance was measured in duplicates using the Tecan spectrometer at a wavelength of 405 nm.

To analyse the proliferation of RAW 264.7 on the surface of the cement specimens (*n* = 4), the DNA concentration was detected. Therefore 20 µl lysate were added to 180 µl of the PicoGreen solution (1:800 PicoGreen reagent dilution with TE buffer; Invitrogen, Karlsruhe, Germany, Cat. P7589) in black 96-well plates and measured it in duplicates using the Tecan spectrometer (extinction 485 nm, emission 535 nm).

The mean value and standard deviation of the quantifiable data were determined from two measurements of each sample. A *t*-test was used for statistical analysis.

Osteoclast differentiation was visualised using a commercial TRAP staining kit (Sigma, Steinheim, Germany, Cat No. 387). To investigate this, RAW 264.7 cells were incubated on the samples with 100 µl of fixing solution for 30 s. The cells were then rinsed twice with deionized water and incubated with 100 µl of staining solution for 60 min. After rinsing the samples again, they were dried and analysed using a stereomicroscope (Discovery.V20, Zeiss, Germany).

To examine the cell structure, a scanning electron microscope (SEM) was used. The RAW 264.7 cells were fixated with 6% glutaraldehyde for 15 min at 0 °C. The samples were then dehydrated using an ascending series of ethanol 30, 50, 70, 90 and 100% for 30 min each. The latter was repeated five times, followed by drying with hexamethyldisilazane (HMDS) for 15 min. The specimens were then sputtered with a 4 nm platinum layer and scanned using a Crossbeam 340 (Zeiss, Oberkochen, Germany) at an acceleration voltage of 5.0 kV and a magnification of 1000-fold.

### ICP-mass spectrometry

The degradation of the cements was analysed by collecting the medium of differentiated RAW 264.7 cells incubated on the surfaces of the cement specimens. Accordingly, the medium of cements that were immersed without cells was also collected as a reference. Samples (*n* = 4) were collected on days 3, 6, 8, 10, 13 and 15 when the medium was changed in the biocompatibility experiments and stored at −80 °C. The ion concentrations of Ca and P were quantified using inductively coupled plasma mass spectrometry (ICP-MS iCAP RQ, Thermo Fisher Scientific, Waltham, USA, Cat. No. BRE731416) against standard solutions of Ca(NO_2_)_2_ (Merck, Darmstadt, Germany, Cat. No. 170308) and H_3_PO_4_ (Merck, Darmstadt, Germany, Cat. No. 170340). Passive resorption was measured by analysing the reference eluates, whereas total resorption was measured using the eluates under the influence of RAW 264.7. The active resorption was calculated by subtracting the passive resorption from the total resorption.

## Results

### Phase analysis

**Fig. 1:**
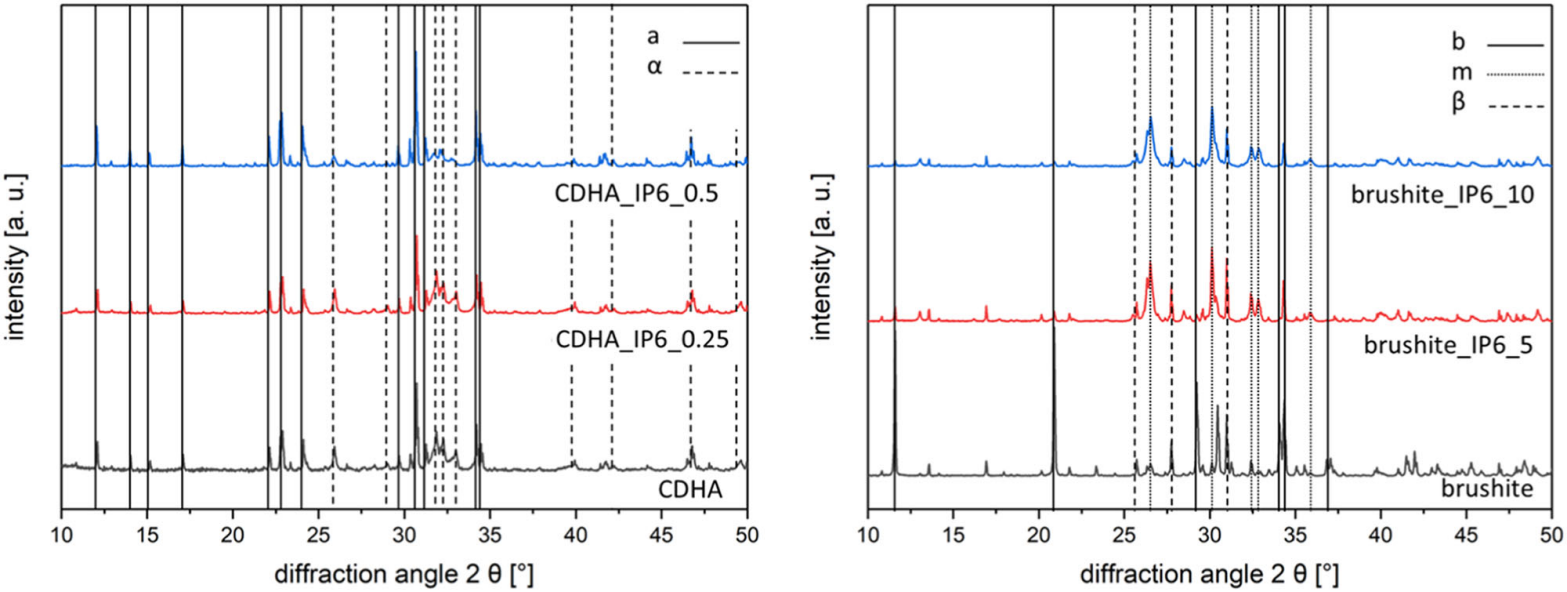
Diffraction pattern of the phase composition of CDHA with 0, 0.25 or 0.5 wt% IP6, brushite with 0, 5 or 10 wt% IP6 (a apatite, α α-TCP, b brushite, m monetite, β β-TCP). The patterns were calculated using TOPAS 4.2 software (Bruker AXS, Karlsruhe)

Phase analysis was carried out to determine the phase fractions of the specimens. The analysis of the cements was conducted after 7 d of hardening with XRD, as shown in Fig. 1. In brushite cements the three phases brushite, monetite and β-TCP where detected. In comparison, brushite cements produced with IP6 had additional peaks of monetite, the anhydrous form of dicalcium phosphate. The ratio of β-TCP was the highest in those cements produced with citric acid.

In all apatite cements CDHA and α-TCP were detected. The phase composition of these cements did not show any significant difference.

### pH value

**Fig. 2:**
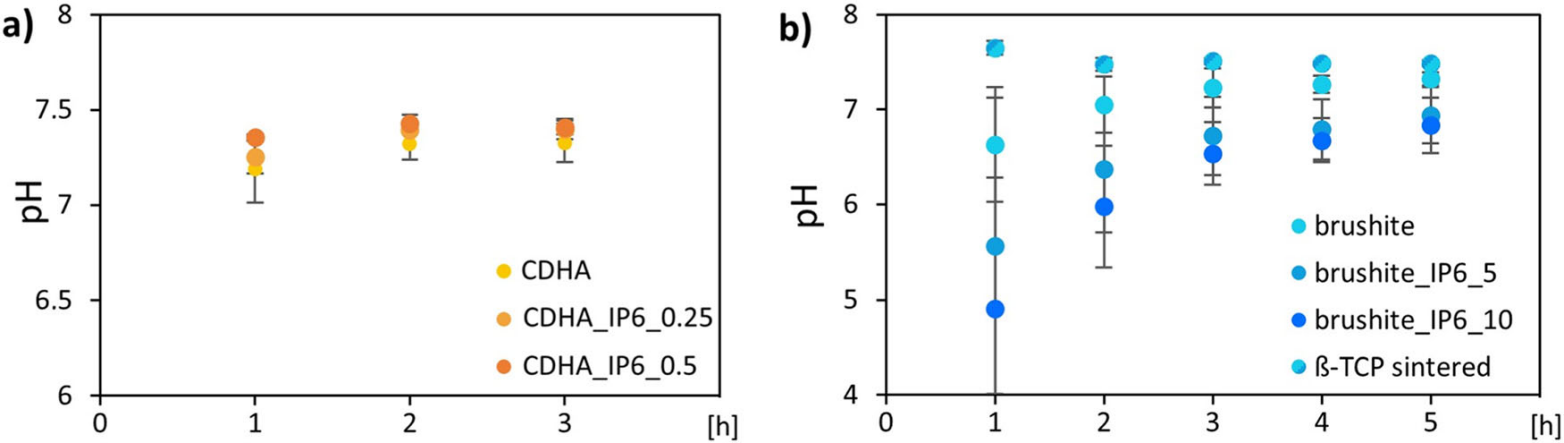
pH profile of the washing solution in which the cements of (**a**) CDHA with 0, 0.25 or 0.5 wt% IP6, (**b**) brushite with 0, 5 or 10 wt% IP6 and sintered β-TCP were placed. The error bars represent the standard deviation (*n* = 3)

The pH profiles of the washing solutions of calcium phosphate cements with different concentrations of IP6 were performed in preparation for biological testing to investigate physiological pH values. They are illustrated in Fig. 2.

The washing solution of all apatite cement specimens showed nearly neutral pH values in the beginning. After 3 h of washing the cements showed pH values of CDHA 7.33 ± 0.10, CDHA_IP6_0.25 7.40 ± 0.05 and CDHA_IP6_0.5 7.41 ± 0.04.

For the brushite specimens more washing cycles were required. The starting pH of each composition was lower due to setting under acidic conditions. While the brushite specimens produced with citric acid started with a pH of 6.63 ± 0.6, the addition of IP6 showed lower pH values of brushite_IP6_5 5.57 ± 1.56 and brushite_IP6_10 4.91 ± 1.37. After 10 cycles of washing only citric acid as setting retarder exhibited a neutral pH of 7.32 ± 0.07, whereas in the washing solution of IP6 cements lower pH values were observed with brushite_IP6_5 of 6.94 ± 0.29 and brushite_IP6_10 of 6.83 ± 0.29. In contrast, sintered β-TCP had an initial basic pH value of 7.65 ± 0.07, which continuously decreased to 7.47 ± 0.02.

### Compressive strength

**Fig. 3:**
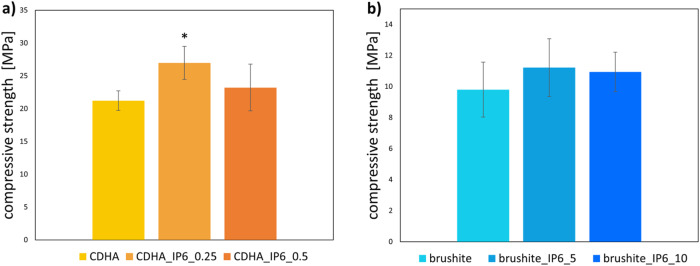
Compressive strength of cuboid (**a**) CDHA and (**b**) brushite specimens with different formulations of IP6 stored for 7 d at 37 °C and 100% humidity. The error bars represent the standard deviation. *p < 0.05 compared to control (*n* = 8)

Figure 3 compares the compressive strength of the different formulations of the cement pastes. Concerning the influence of IP6 in the produced specimens, the highest compressive strength in CDHA cements was observed after hardening for 7 d in CDHA_IP6_0.25 with 26.99 ± 2.52 MPa. It showed to be significantly higher than the CDHA reference (21.22 ± 1.49 MPa). Indeed, higher amounts of IP6 didn’t enhance the compressive strength further but demonstrated comparable values of 23.22 ± 3.56 MPa.

On the other hand, the brushite specimens showed relatable results. Likewise, the smaller amount of IP6 in brushite_IP6_5 yielded the highest compressive strength of 11.22 ± 1.86 MPa, and the higher amount of IP6 in brushite led to a reduction of physical strength to 10.94 ± 1.27 MPa.

### Osteoblastic like cells

**Fig. 4:**
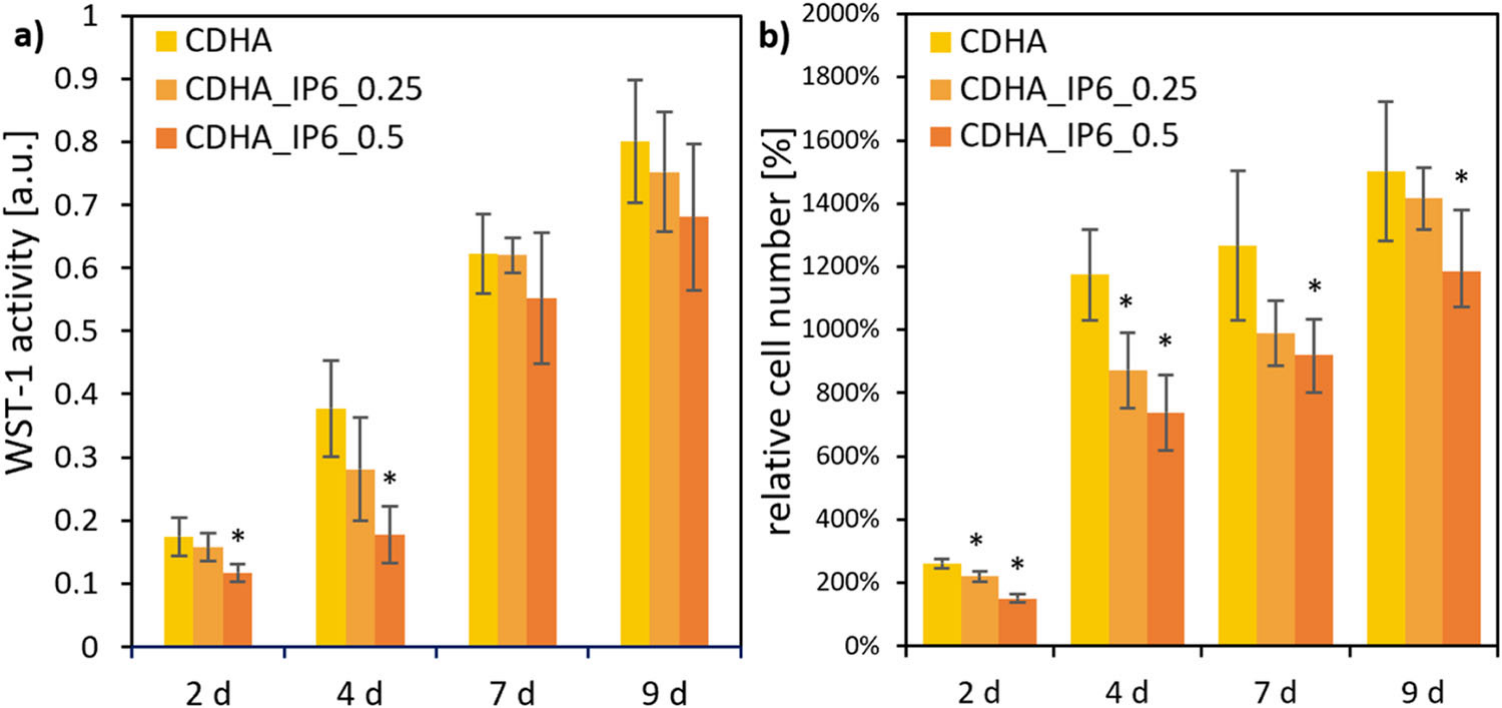
Cell activity (**a**) and cell number (100% at 50,000 cells) (**b**) of MG-63 on CDHA specimens with regard to different wt% of IP6. The error bars represent the standard deviation. *p < 0.05 compared to control (*n* = 4)

Over the complete testing period of 9 d no significant difference was detected for the WST-1 activity of the osteoblastic-like cell-line MG-63 on the surfaces of the tested CDHA specimens (Fig. 4). In comparison only the CDHA probes produced with a higher amount of IP6 showed a significantly lower cell activity at the first two points of measurement, which adjusted to the end of the measurement. This was confirmed by the comparable results of the relative cell numbers (the initial added cell number of 50,000 cells was set at 100%). Over the complete period CDHA_IP6_0.5 had significantly lower cell numbers than the control. At the first two measuring points, CDHA_IP6_0.25 exhibited significantly lower cell numbers.

**Fig. 5:**
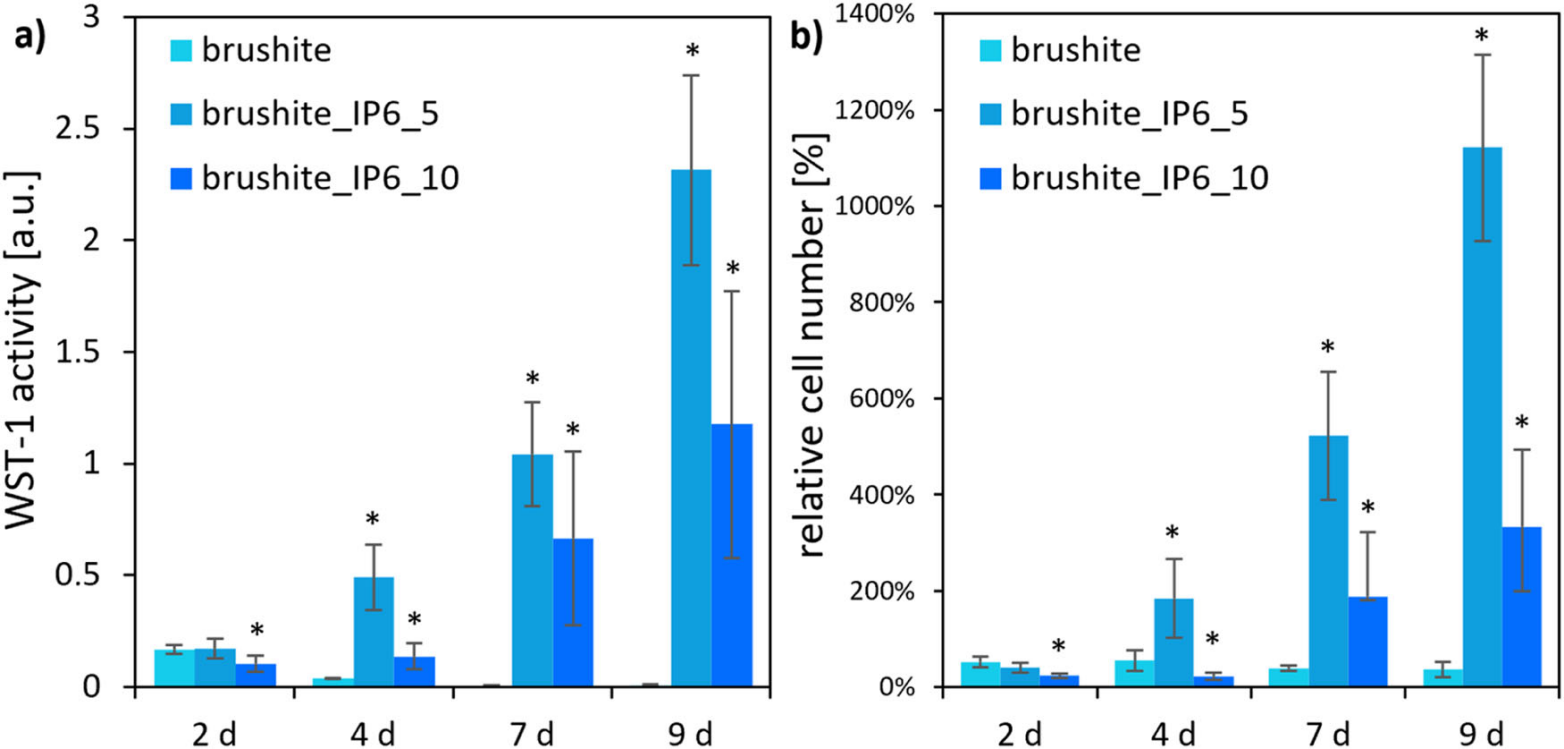
Cell activity (**a**) and cell number (100% at 50,000 cells) (**b**) of MG-63 on brushite specimens with regard to different wt% of IP6. The error bars represent the standard deviation. *p < 0,05 compared to control (*n* = 4)

Likewise, the MG-63 cells on the brushite specimens (Fig. 5) showed a higher increase in cell activity and relative cell number when adding a lower amount of 5 wt% than of 10 wt% IP6. Both showed a significant higher WST-1 activity of the cells compared to the control after day 4 of cultivation. The cells on the control cement composed with citric acid on the other side exhibited a distinct decline of cell activity over the testing period. The highest cell numbers for MG-63 cells on brushite_IP6_5 between were registered day 4–9. The control cement resulted in a decrease in cell numbers to less than half of the initial count. However, the addition of IP6 led to an increase of over 11 times for brushite_IP6_5 and over 3 times for brushite_IP6_10 of the originally seeded cell count.

### TRAP activity quantitative

**Fig. 6:**
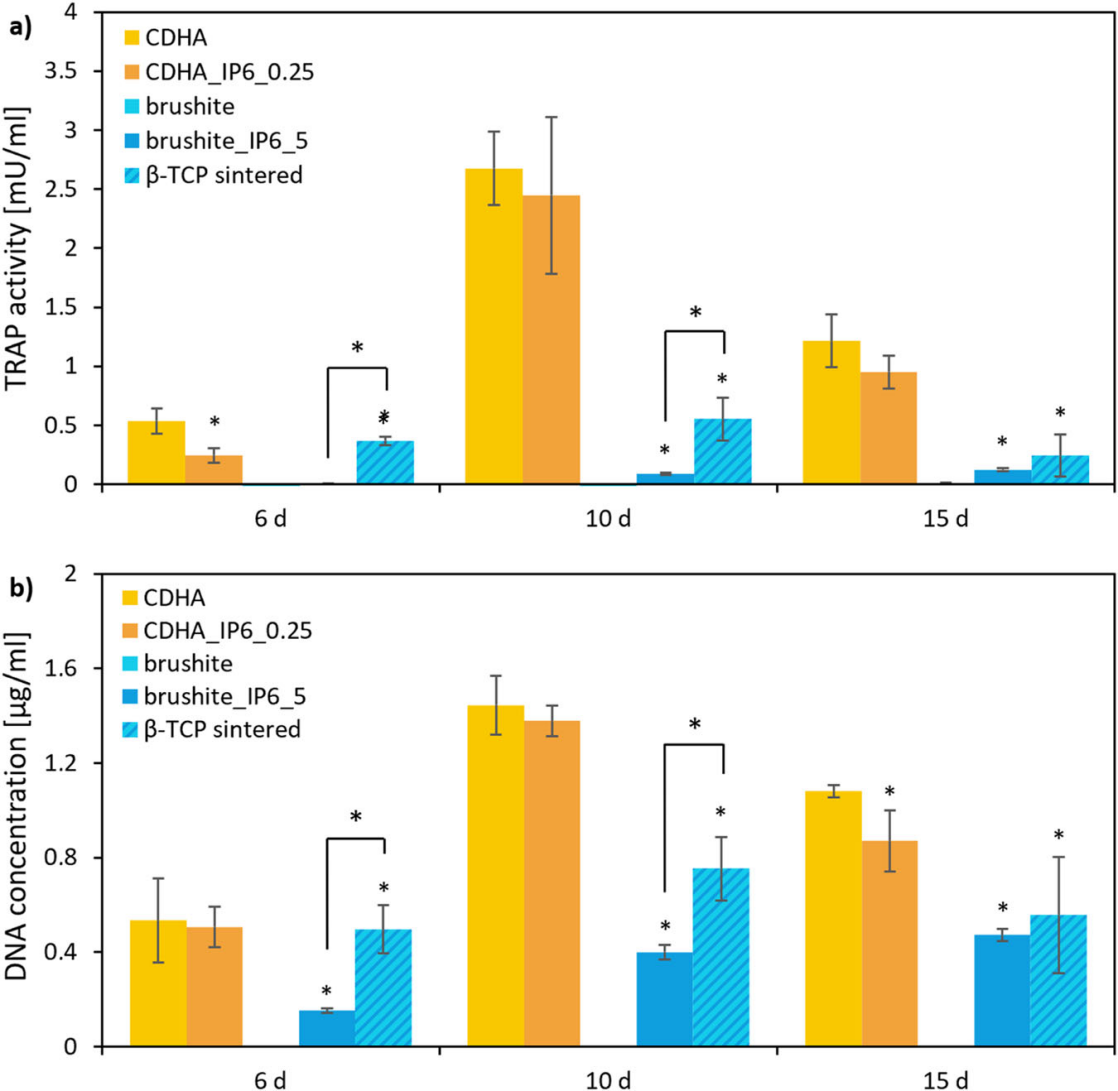
TRAP activity (**a**) and DNA concentration (**b**) of RAW 264.7 cells incubated with 50 ng/ml RANKL on calcium phosphate cements with different IP6 concentrations over 15 d. The error bars represent the standard deviation. *p < 0,05 compared to control (*n* = 4)

A well-established way to investigate the cytocompatibility of cements with osteoclastic cells is to culture RAW 264.7 cells on their surfaces. To differentiate the macrophage cell line, 50 ng/ml RANKL was added to the culture medium. To quantify the differentiation and activity of the RAW cells the activity of the specific enzyme TRAP and the DNA concentration was measured. Figure 6 displays the results.

The control CDHA cement and CDHA_IP6_0.25 both showed an initial increase in TRAP activity and DNA concentration, which showed a discreet levelling until day 15. Throughout the testing period, the specimens formed with IP6 exhibited comparable properties to the control.

On the surface of the brushite cement with citric acid as a setting retarder, there was almost no cell growth or activity detected. In contrast, the two other specimen types formed with β-TCP reached significantly higher TRAP activity and DNA concentration compared to the control. Indeed, the sintered β-TCP initially showed significantly higher levels in cytocompatibility than brushite_IP6_5 which aligned till day 15.

### TRAP staining and SEM

**Fig. 7:**
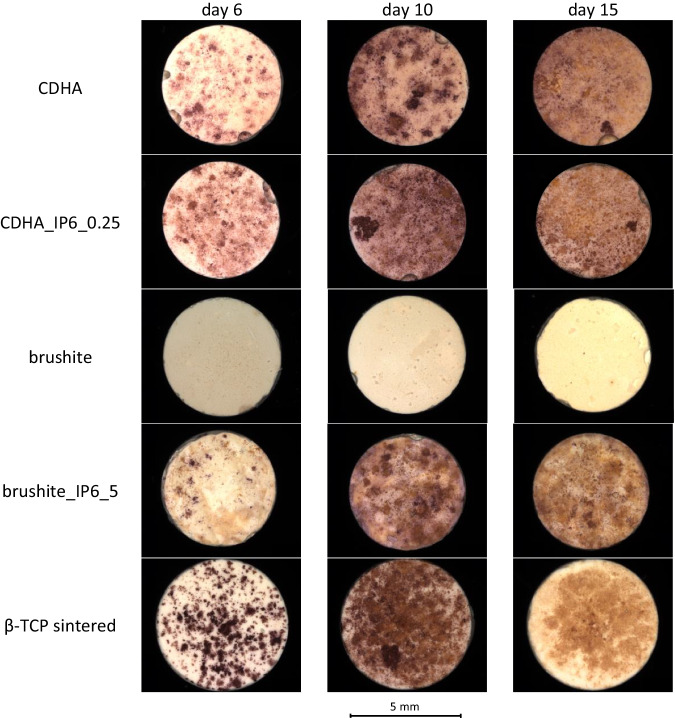
Qualitative visualisation of TRAP via TRAP staining of RAW 264.7 differentiated with RANKL on calcium phosphate cements. Differentiation can be seen for calcium phosphate cements with and without IP6 and for sintered ß-TCP over a culture period of 15 d. For scale a bar of 5 mm is specified

**Fig. 8:**
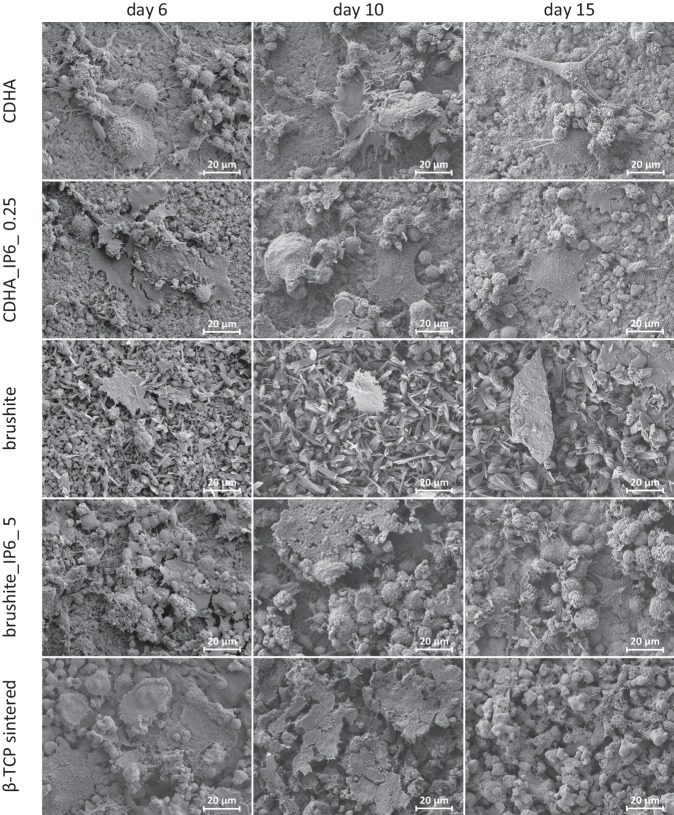
SEM images of RAW 264.7 differentiated with RANKL on the surface of the various calcium phosphate cements at day 6, 10 and 15. The images are made at 1000× magnification and given with a scale of 20 µm

The differentiation of RAW 264.7 cells into polynuclear osteoclastic cells by adding RANKL was verified by TRAP staining (Fig. 7) and SEM (Fig. 8) of the cement surfaces. On brushite specimens with citric acid, only isolated small staining patterns were observed and in SEM, only the cement structure and cell detritus were determined. The other surfaces exhibited an evolution from growing violet cell clusters on day 10 to brownish-violet cross-surface staining after 15 d. The sintered β-TCP displayed a declining staining in the last cultivating period. In the SEM analysis, only cell residues were detected. Additionally, SEM imaging revealed fused cells on CDHA, CDHA_IP6_0.25 and brushite_IP6_5 after 6 d of culture. After 10 and 15 days, the morphology transitioned to an osteoclastic phenotype characterized by a typical flat or elongated shape with fimbriated features and filopodia [26, 27].

### Degradation

The degradation of the cements was tested by preserving the used culture medium. This was carried out without cells as passive degradation and with differentiated RAW 264.7 cells as total degradation. The medium was then analysed by ICP.

**Fig. 9:**
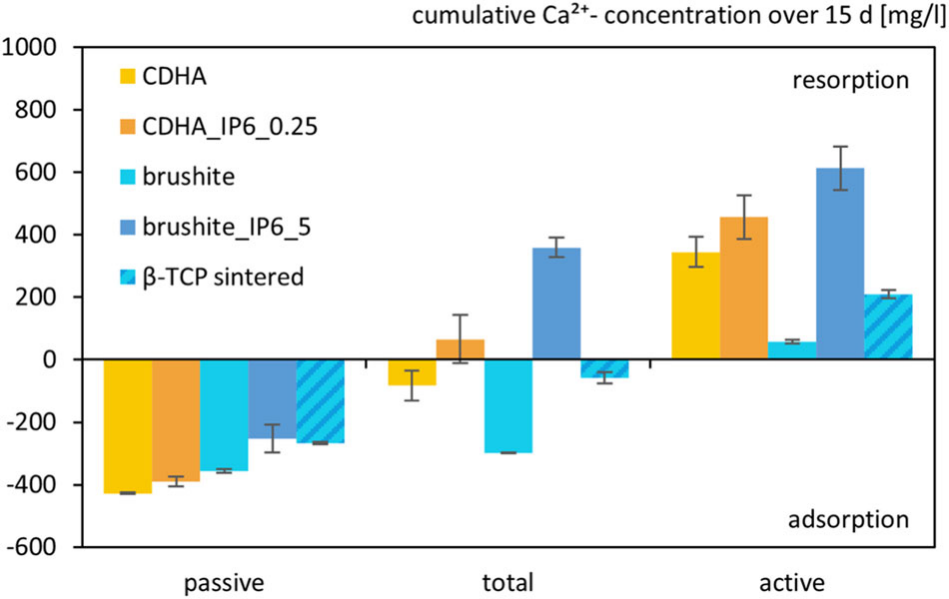
Cumulative Ca^2+^ ion concentration due to their release by passive, total and active resorption of calcium phosphate cements for 15 d. The specimens were incubated without cells for passive resorption, with cells for total resorption and the difference between those two was formed for active resorption. The error bars represent the standard deviation (*n* = 4)

Regarding the calcium concentration (Fig. 9) the passive degradation of control cements showed higher adsorption of ions on the specimens than the cements with IP6 as setting retarder and the sintered β-TCP. It is noteworthy that all specimens revealed a higher degradation when seeded with RAW 264.7 cells. Though only in the two specimens formed with IP6 a resorption of calcium could be demonstrated with 360 mg/l for brushite_IP6_5 and 65 mg/l for CDHA_IP6_0.25. Overall, the addition of cells resulted in calcium resorption, as displayed in the active degradation.

**Fig. 10:**
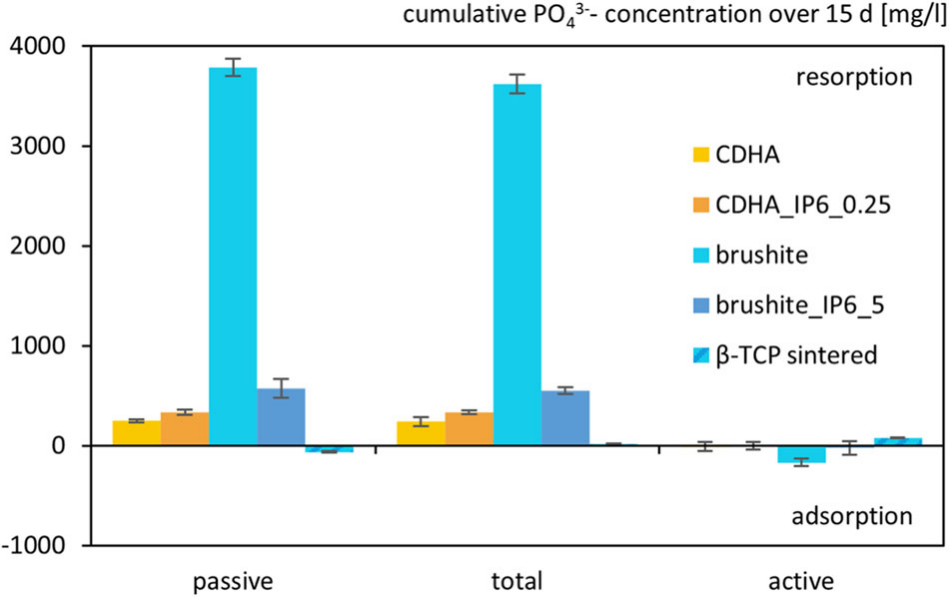
Cumulative PO_4_^3−^ ion concentration due to their release by passive, total and active resorption of calcium phosphate cements for 15 d. The specimens were incubated without cells for passive resorption, with cells for total resorption and the difference between those two was formed for active resorption. The error bars represent the standard deviation (*n* = 4)

Regarding the degradation of phosphate (Fig. 10) for most specimens no difference was observed between the total and passive degradation. Except for the sintered β-TCP all specimens exhibited a distinct phosphate resorption. In this regard, the brushite cement had the highest cumulative phosphate concentration of more than 3.600 mg/l. Moreover, the total degradation for these specimens was lower than the passive degradation which resulted in an adsorption for the active degradation.

## Discussion

The relatively short setting time of calcium phosphate cements such as CDHA and particularly brushite cements restricts the clinical use of these substitute materials. Hence, setting retarders like citric acid, pyrophosphate or sulphuric acid are used to extend this time interval [28]. A comparatively new approach is the utilisation of IP6. Meiniger et al. could show that the addition of 0.5 M citric acid could prolong this period to more than 5 min and 0.1 M IP6 to approximately 4 min for brushite cements [15]. Likewise, Weichold et al. showed that a low viscosity for CDHA was prolonged for more than 5 min, however a higher quantity of IP6 did not lead to any further extension [24].

The general phase composition was very similar in both cements. However, the addition of IP6 resulted in lower TCP peaks. The resulting increased formation of monetite in brushite cements correlated with the amount of IP6, which is supported by Hurle et al. [25]. This phase change could be explained by chelation of citrate and formation of amorphous compounds.

Moreover, the compressive strength of the cements is improved by the use of setting retarders. In the present study this could be confirmed for both cement types. The highest compressive strength was achieved by respectively the lowest quantity of IP6, while a higher quantity led to a reduction of the compressive strength (Fig. 2). An increase in the previously described amount of monetite relative to brushite is consistent with the reduction of brushite in favour of monetite at higher IP6 concentrations. On the other hand, the increase in strength with CDHA_IP6_0.25 appears surprising, as no setting retarder such as citrate was used in the control, which could alter the composition of the cement. Whilst for CDHA we expect improved strength due to the slightly prolonged setting reaction and therefore improved entanglement of the crystals, a further increase in IP6 will result in incomplete hardening of the cement and lower compressive strength. This is consistent with the findings of Weichhold et al., who demonstrated that small amounts of IP6 increased the compressive strength and in addition the injectability of CDHA cements. However, no further increase in the effect was achieved with higher concentrations of IP6 [24].

Even though good physical characteristics arise from adding citric acid [29, 30] there is mounting evidence that the biological properties of this setting retarder are inferior to others. In specimens formed with citrate a loss of cell attachment could be observed [16]. Furthermore, Meininger et al. could demonstrate, that the use of IP6 as a setting retarder in brushite cements, in comparison to citric acid, improved the WST-1 activity of osteoblastic and osteoclastic cells [15]. The results of our study support these findings. Instead of using WST-1, we used the more specific TRAP activity to quantify the activity of the osteoclastic RAW 264.7 cells. In addition we could show, that a higher amount of IP6 leads not only to a significantly lower compressive strength but also to a lower WST-1 activity and cell number of osteoblastic cells (Fig. 4). On the surface of the CDHA cements, the cells of the osteoblast-like MG63 displayed a much more homogeneous image. Over the entire testing period with MG63, the CDHA_IP6_0.25 demonstrated comparable cell activity and cell number to the CDHA control. As with the brushite specimens, the CDHA_IP6_0.5 however had significantly poorer biological properties. On the one hand this can be attributed to the delayed reaction and remaining α-TCP and the increased formation of IP6 in the solution on the other. Furthermore, due to the lower strength of the CDHA cement at higher concentrations of IP6, the adherence of the cells could be reduced.

The used monocytic RAW 264.7 is an established cell line for sintered or moulded cement specimens and can be differentiated to osteoclastic cells by adding RANKL [2, 31–33].

Like Meininger et al. [15] in the osteoclastic testing, we only could detect very low cell activity for brushite specimens formed with citric acid. Although we measured the DNA concentration to identify even small cell clusters, we only found negligible amounts of DNA on brushite specimens formed with citric acid. In contrast the activity and the DNA concentration of brushite_IP6_5 showed a continuous increase. Additionally, we compared these moulded brushite cements with sintered β-TCP, which initially exhibited not only higher TRAP-activity, but also elevated DNA concentrations. However, after 15 d, there was a distinct decrease of these cytocompatibility markers on sintered β-TCP, so that there was no significant difference compared to brushite_IP6_5. As in the osteoblast-like cell tests, the RAW cells displayed a comparably high TRAP-activity and DNA-concentration. The control cement tended to show slightly higher parameters. In comparison with the cements formed with β-TCP, the CDHA cements had 2-3 times higher DNA-concentrations, but significantly higher TRAP-activities. These findings, which occurred especially at the first measuring points, indicate that not only the cell proliferation, but also the differentiation of osteoclastic cells is more successful on CDHA than on brushite cements, which is supported by the findings of Detsch et al. [34].

The differentiation of murine RAW 264.7 cells into multinucleated osteoclasts was analysed by qualitative TRAP staining (Fig. 6) and SEM (Fig. 7). The surfaces of CDHA cements showed a uniform cumulative staining over the whole culture period, which suggests a differentiation already on day 6. The SEM also revealed an earlier differentiation of murine cells into giant multinucleated cells for both CDHAs, compared to the brushite specimens. Moreover, distinct smaller osteoclasts formed on the surface of cements with β-TCP. The brushite specimens showed a varying staining. On samples with citric acid, only microscopic staining was detectable, whereas the staining on IP6 samples demonstrated a differentiation at the latest on day 10. Comparable findings were found by Meininger et al. [15] through the characterisation of brushite cements. Besides, the sintered β-TCP specimens displayed initially similar differentiation to CDHA in TRAP staining, however there was a significant decrease in the stained cell clusters towards the end of the culture period. In addition to the quantitative analysis, these results are also reflected in the SEM examination, where a reduction of osteoclasts could be found on the surface of sintered β-TCP after 15 d. We assumed that the increase of calcium concentration through osteoclasts induced a detachment [35] of the osteoclasts, which were not able to form lacunae on the sintered surface of the β-TCP [34] (Detch et al.) to sustain the cell adhesion. With regards to the degradation of the sintered β-TCP, the concentration of Ca^2+^ or PO_4_^3−^ in the medium also stagnated during this period. Clarke et al. could also demonstrate, that the pH value of the culture medium on β-TCP dropped to 6.9 after 8 d of culturing RAW cells, but subsequently rose back to the neutral range [36]. This reflects the results on the inconsistent biological behaviour of β-TCP over the last years [37].

Concerning the degradation of the other specimens, interestingly enough, both of the cements produced with IP6 presented a net resorption of Ca^2+^ cumulating over the testing period, when cultured with osteoclastic RAW 264.7 cells. These findings in comparison to the control cements suggest a better substitution with new bone formation in vivo.

Furthermore, all of the tested cements except the sintered β-TCP showed a PO_4_^3−^ release in total and passive resorption, which in context with an adsorption of Ca^2+^ is associated with a conversion to more complex calcium phosphate cements. This shift is characteristic of brushite cements, which is reflected in this study by the brushite cement with citrate as a setting retarder. The brushite formed with IP6, on the other hand, exhibited the lowest Ca^2+^ adsorption of all the cements in a passive setting and a comparable PO_4_^3−^ release to CDHA cements. This indicates a much lesser tendency to form phases with a higher Ca:P ratio than other brushite cements [38–40].

## Conclusion

Osteoblast-like cells (MG63) and murine cells (RAW 264.7) were cultivated on calcium phosphate cements prepared from α-TCP or β-TCP to which the setting retarder IP6 was added. The differentiation of RAW 264.7 was carried out by adding 50 ng/ml RANKL. Besides the improved physical properties such as higher compressive strength and extended processing time, this study revealed a similar cell proliferation and activity for IP6 in CDHA cements and even significantly higher biocompatibility for brushite cements than when citric acid was used. Remarkably, the increase of IP6 did not improve the mechanical or biological properties of the specimens. IP6 also resulted in better resorbability of the cements both with and without osteoclast-like cells in vitro. Overall, IP6 is an auspicious additive that could significantly improve the clinical utilisation of calcium phosphate cements as bone graft substitutes.
